# A comparative study of limb load on the healthy side of above-knee amputees after reconstruction of assistive walking devices

**DOI:** 10.1097/MD.0000000000041684

**Published:** 2025-04-11

**Authors:** Lingjie Zeng, Yitian Wang, Minxun Lu, Yong Nie, Yong Zhou, Li Min, Xiangdong Zhu, Chongqi Tu

**Affiliations:** a National Engineering Research Center for Biomaterials, Sichuan University, Chengdu, China; b Sports Institute, Chengdu University of Technology, Chengdu, China; c Department of Orthopedics, West China Hospital, Sichuan University, Chengdu, China.

**Keywords:** above-knee amputees, assistive walking devices, comparative study, limb load

## Abstract

Patients with above-knee amputations often experience postoperative secondary diseases in their unaffected leg, including reduced bone density, muscle atrophy, and osteoarthritis. These conditions arise due to altered limb load resulting from the amputation. Assistive walking devices offer additional support and can modify limb load. To assess the impact of different assistive devices on limb load in the unaffected leg, we conducted a study with 13 above-knee amputation patients and thirteen healthy individuals. Participants walked at a self-selected speed while using a prosthetic, single crutch, or double crutches. Gait analysis was performed using Qualisys motion capture and force plates, and kinematic and kinetic data were analyzed using custom OpenSim software. Our results indicate that the prosthetic group closely resembles the healthy group but exhibits differences in knee joint adduction moments and knee joint contact forces. Among the groups, the single-crutch group bears the highest load, while the double-crutches group has the lowest load, even lower than the healthy group. Bio-inspired prosthetics are recommended for long-term use, but optimizing kinematic symmetry through rehabilitation methods and prosthetic modifications is essential. Prolonged use of crutches should be minimized to reduce stress concentration, and resistance training is a suitable strategy.

## 1. Introduction

Above-knee amputation is a common surgical procedure in clinical practice, often resulting from trauma, bone tumors, peripheral vascular diseases, or diabetes.^[1,2]^ They account for a significant proportion of all amputation surgeries, approximately 54%.^[3]^ Humans are bipedal creatures, and limb loss can disrupt walking. Therefore, postamputation patients use various walking aids to regain ability and ensure basic quality of life. However, regardless of the type of assistive walking device used, above-knee amputees are at higher risk of developing secondary conditions on the healthy side (such as reduced bone density,^[4]^ muscle atrophy,^[5]^ and osteoarthritis^[6]^) compared to below-knee amputees and healthy individuals.^[7,8]^

The occurrence of secondary complications is closely linked to limb loading following amputation. Reduced bone density^[9]^ in the residual limb and muscle atrophy stem^[10]^ from adaptive changes due to insufficient load. These conditions often coexist and correlate with the severity of fractures.^[9]^ The primary cause of these complications is limb unloading, which occurs during postoperative limb suspension and in microgravity environments.^[11]^ Recent research by Finco,^[7]^ summarizing 27 articles on muscle and skeletal adaptations after amputation, revealed that over 80% of patients experience varying degrees of reduced bone density and muscle atrophy. The period from 6 months to 1 year after surgery is particularly sensitive to these changes. Regular use of isokinetic resistance exercises can help mitigate this process. Additionally, osteoarthritis arises from asymmetric compensation resulting from prolonged high loads.^[12]^ Notably, the knee adduction moment serves as a sensitive indicator of knee osteoarthritis,^[13]^ showing a clear correlation with external adduction moments.^[8]^

From a biomechanical perspective, limb loading following amputation should closely approximate that of a healthy individual. Both excessive and insufficient loading can contribute to secondary complications, impacting patients’ postoperative quality of life. After amputation, patients have 2 primary strategies for modifying limb loading: rehabilitation training^[14]^ and the utilization of assistive walking devices.^[15]^ As above-knee amputees, selecting an appropriate walking aid that aligns with the physiological limb loading of healthy individuals becomes crucial. Furthermore, identifying specific indicators for rehabilitation targets and exploring potential optimizations for assistive walking devices are essential research questions. However, currently, there is a lack of dedicated studies addressing the limb loading patterns of above-knee amputees when using various walking aids.

Therefore, we conducted biomechanical gait tests on commonly used assistive walking devices (crutches, canes, and prostheses) for above-knee amputees using a custom OpenSim model. We compared the results with a healthy control group. This study had 2 main objectives: (1) to elucidate the impact of various assistive walking aids on the load distribution in the healthy contralateral lower limb, and (2) to compare the lower limb loading between different assistive walking devices and the healthy control group. The findings from this research will provide data support for clinical rehabilitation strategies and assistive device selection.

## 2. Methods

### 2.1. Participants

In this study, 13 patients (9 males, 4 females) who underwent above-knee amputation (6 left; 7 right) were chosen (mean ± SD: age, 59.2 ± 7.8 years; height, 1.75 ± 0.06 m; weight, 68.15 ± 7.59 kg; body mass index, 22.09 ± 1.17 kg/m^2^). All patients had undergone amputation more than 18 months ago and began prosthetic training approximately 7 to 9 weeks postamputation. They are currently able to independently use prosthetics without assistive devices, have no recent history of falls, and underwent 2 to 6 weeks of crutch training during the rehabilitation period.

A control group of 13 healthy volunteers was also selected (mean ± SD: age 53.5 ± 6.4 years; height 1.76 ± 0.1 m; weight 70.02 ± 9.21 kg; BMI 24.3 ± 1.8 kg/m^2^). All participants had no additional neurological or physical defects affecting balance or gait. The procedures of this study complied with the declaration of Helsinki. All procedures were approved by the Ethics Review Committee of West China Hospital, and written informed consent was obtained from all the subjects for the publication, including the use of any accompanying images and case details.

### 2.2. Equipment

Kinematic data were collected at 290 Hz using a 10-camera motion analysis system (Oqus300, Qualisys, Gothenburg, Sweden). Each subject walked at a self-selected pace on a 12 m walkway. Ground reaction forces were recorded with 2 force platforms arranged in a line, (Bertec, Columbus); Reflective markers were placed on the anatomical landmarks, the prosthetic is similar to a Cappozzo, Catani, Della Croce, and Leardini model (CAST model), and the 2 crutch groups have removed the marker below the hip joint of the amputated limb on the basis of CAST model.

Marker trajectories were filtered using a Butterworth filter with a cutoff frequency of 6 Hz. Inverse dynamics were filtered by a zero-lag fourth-order Butterworth filter with a cutoff frequency of 6 Hz.

### 2.3. Study design

**Figure 1.**
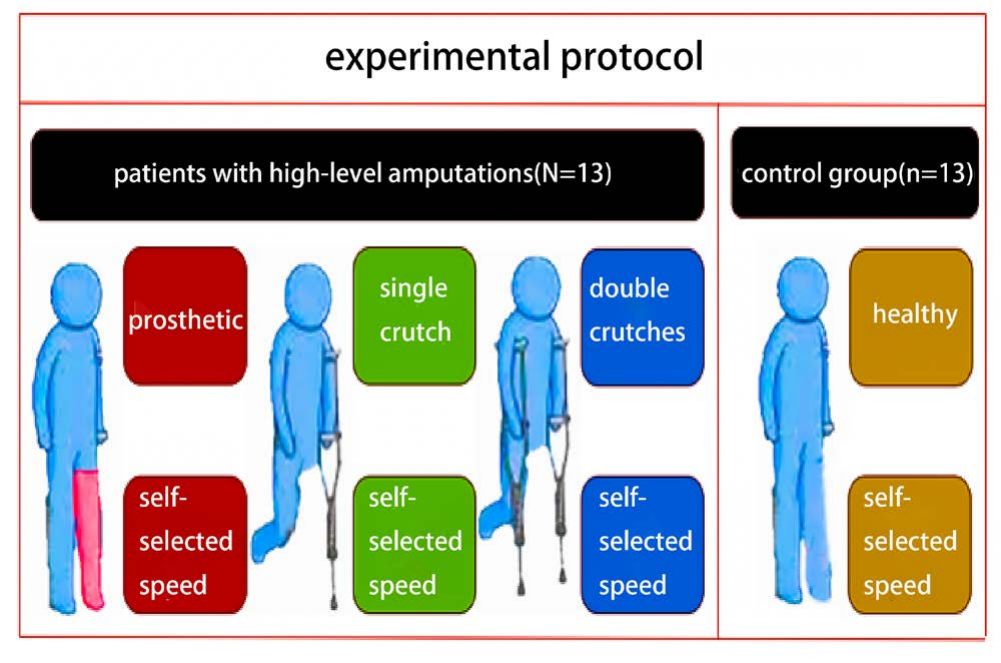
Experimental protocol for prosthetic, single crutch, double crutches, and healthy groups.

Each patient used 3 different walking aids (prosthesis; single crutch; double crutches) to walk on the sidewalk at a self-selected speed (Fig. 1). The control group volunteers also walked on the sidewalk at a self-selected speed. The marker set was changed accordingly. Subjects were asked to keep their backs as straight as possible and to look straight ahead. The crutches used were underarm crutches, and the prostheses were the ones patients regularly used. These were mechanical prostheses, with no additional power source.

### 2.4. Model establishing

#### 2.4.1. Define the torso

Opensim scaled the torso with head and fifth sacral vertebra (V. Sacral)point; the V.Sacral point was defined through Mokka (motion kinematic & kinetic analyzer) by creating an average marker between right posterior superior iliac spine (R_IPS) and left posterior superior iliac spine (L_IPS).

#### 2.4.2. Prosthetic modeling

We used SolidWorks to construct computer-aided design (CAD)models of a generibc socket, pylon, and foot, and scaled the prosthetic CAD model to actual size. By aligning the center of rotation of the prosthetic knee joint with the knee joint center of the OpenSim model, the CAD model of the prosthesis was inserted into the OpenSim2392 model.

**Figure 2.**
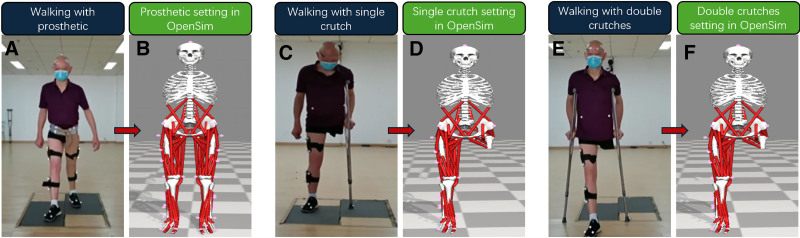
(A) Maker set in prosthetic group (B) maker set of prosthetic group in OpenSim model (C) maker set in single-crutch group (D) maker set of single-crutch group in OpenSim (C) maker set in double crutch group (D) maker set of double-crutches group in OpenSim model.

The OpenSim 2392 model was used to analyze the healthy group; for the other 3 walking assistance device groups, the OpenSim 2392 model served as the basic model. The original marker of the 2392 model was replaced with the experimental marker, and corresponding changes were made in the related code at the same time (Fig. 2). The prosthetic mass was used instead of the infected limb mass on the prosthetic model, and the infected limb mass was set to zero on the crutch model. Dynamic analysis involved only the healthy leg.

### 2.6. Data processing

#### 2.5.1. Joint moment

The joint moment is expressed in the stance phase and normalized to body weight. The first and second peaks of the adduction moment were defined as the maximum value of the adduction moment profile in the first and second halves of the stance phase, respectively.

The moment waveform was integrated using matrix laboratory (MATLAB) to obtain the moment impulse, which is calculated using the following equation: moment impulse $=\overset{b}{\underset{a}{\int }}moment(t)dt$.

#### 2.5.2. Joint reaction forces

The joint reaction forces is expressed in the stance phase and normalized to body weight. The first and second peaks of the joint contact forces were defined as the maximum value of the joint contact forces in the first and second halves of the stance phase, respectively.

The moment waveform was integrated using MATLAB to obtain the joint contact forces impulse, which is calculated using the following equation: moment impulse $\overset{b}{\underset{a}{=\int }}jointcontactforce(t)dt$.

### 2.8. Statistical analysis

Standard descriptive statistics followed by repeated-measures analysis of variance were used in the statistical analysis. The level of statistical significance was set at *P* < .05. Analyses were performed using SPSS. Data were expressed as means ± SD. Comparisons were made pairwise using 1-way analysis of variance.

## 3. Results

### 3.1. Gait parameters

Thirteen patients, walked with different walking aids (single crutch, double crutches, and prosthetic), respectively, the kinetic and dynamic analysis involved only the healthy leg. Thirteen healthy volunteers walked at self-selected speed as the control group (Table 1).

**Table 1:** Gait parameters at the healthy side.

	Single crutch (a)	Double crutches (b)	Prosthetic (c)	Healthy (d)	Comparison
Walking speed (m/s)	0.65 ± 0.05	0.70 ± 0.02	0.73 ± 0.09	1.07 ± 0.17	d > c,b > a
Step length (m)	0.44 ± 0.07	0.5 ± 0.1	0.67 ± 0.06	0.64 ± 0.06	c,d > b,a
Cadence (step/min)	88.24 ± 4.72	83.92 ± 2.52	65.75 ± 5.36	98.15 ± 7.52	d > a > b > c
Step width (m)	0.22 ± 0.02	0.18 ± 0.02	0.15 ± 0.02	0.17 ± 0.02	a > b,d > c
Stance time (% gait cycle)	70.10 ± 4.21	66.47 ± 2.31	68.50 ± 3.08	62.46 ± 3.2	a,c,b > d

Gait parameters are tested at the healthy side in the single crutch, double crutches, and prosthetic groups (n = 13), in the healthy group, gait parameters are tested at the right side (n = 13); all results in comparison have significance (*P* < .01).

The healthy group had the highest walking speed (1.07 ± 0.17 m/s) and cadence (98.15 ± 7.52 steps/min) and the shortest stance time ratio (62.46% ± 3.2%) among the 4 groups. The other 3 groups showed some differences in gait parameters compared to the healthy group. Specifically, the single-crutch group had the lowest walking speed (0.65 ± 0.05 m/s), step length (0.44 ± 0.07 m), and the highest step width (0.22 ± 0.02 m) and stance time ratio (70.10% ± 4.21%).

### 3.2. Joint moment

**Figure 3.**
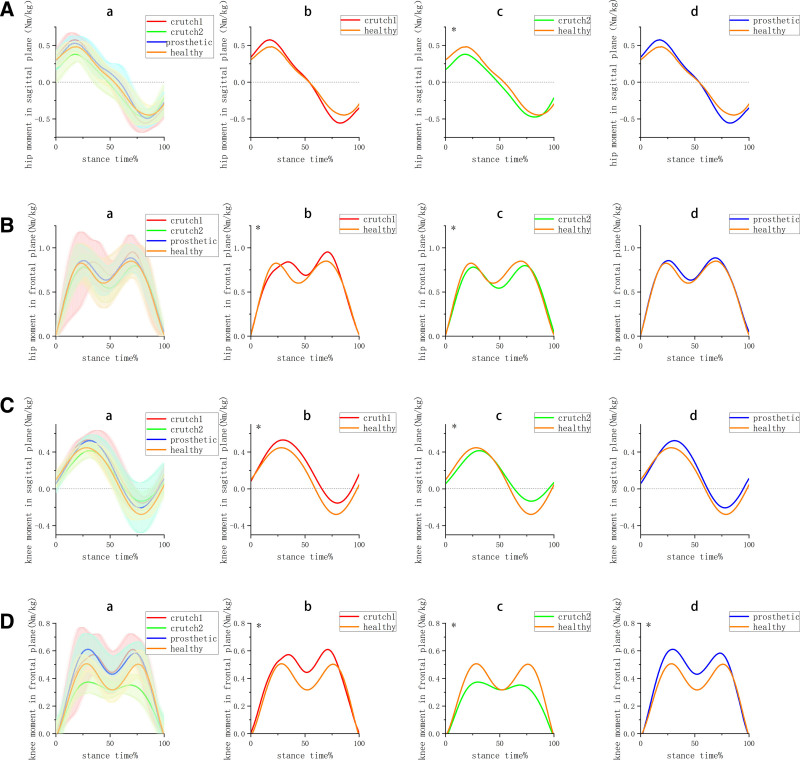
Curves of the joint moment on the sagittal and frontal plane at the healthy side (n = 13); crutch1 is the single-crutch group, crutch2 is the double-crutches group, and prosthetic is the prosthetic group, healthy is the healthy group. (A) hip moment in the sagittal plane (B) hip moment in the frontal plane (C) knee moment in the frontal plane (D) hip moment in the frontal plane. (a) comparison among 4 groups (b) comparison between single-crutch group and healthy group (c) comparison between double-crutches group and healthy group (d) comparison between prosthetic group and healthy group, *means *P* < .01.

In the single-crutch group (Fig. 3), there was significant difference in the hip joint moment at the frontal planes, while there was no significant difference at the sagittal plane when compared to the healthy group. The hip joint moment parameters (Table 2) were generally similar to those of the healthy group, except for the hip peak flexion moment (0.6 ± 0.06 Nm/kg), which was significantly higher than that of the healthy group (0.52 ± 0.05 Nm/kg).

**Table 2:** The joint moment parameters of the healthy side.

	Single crutch (a)	Double crutches (b)	Prosthetic (c)		Healthy (d)	Comparison
Hip peak flexion moment (Nm/kg)	0.6 ± 0.06	0.43 ± 0.08	0.58 ± 0.09		0.52 ± 0.05	a > d > c > b
Hip peak extension moment (Nm/kg)	(−0.59) ± 0.1	(−0.52) ± 0.09	(−0.54) ± 0.12		(−0.5) ± 0.1	
Hip flexion impulse (Nm.ms/kg)	123.01 ± 37.4	69.12 ± 22.2	111.29 ± 31.43		104.94 ± 36.58	a,c,d > b
Hip extension impulse (Nm.ms/kg)	−108.43 ± 35.61	−94.49 ± 29.63	−79.43 ± 19.27		−86.43 ± 34.17	a > c
The first peak of hip adduction moment (Nm/kg)	1.05 ± 0.16	0.81 ± 0.13	0.92 ± 0.12		0.92 ± 0.19	a,c,d > b
The second peak of hip adduction moment (Nm/kg)	1.01 ± 0.17	0.83 ± 0.07	0.95 ± 0.12		0.98 ± 0.16	a,c,d > b
Hip adduction impulse (Nm.ms/kg)	359.36 ± 107.79	315.05 ± 81.78	384.73 ± 85.9		366.62 ± 111.51	a,c,d > b
knee peak flexion moment (Nm/kg)	0.56 ± 0.03	0.43 ± 0.07	0.52 ± 0.04	0.46 ± 0.05		a > c > d,b
knee peak extension moment (Nm/kg)	(−0.19) ± 0.06	(−0.15) ± 0.09	(−0.3) ± 0.07	(−0.31) ± 0.06		│c,d│>│a,b│
Knee flexion impulse (Nm.ms/kg)	129.87 ± 45.66	92.41 ± 25.06	120.82 ± 33.2	110.03 ± 37.34		a,c > b
Knee extension impulse (Nm.ms/kg)	(−22.69) ± 19.16	(−18.47) ± 16.28	(−40.24) ± 10.22	(−44.56) ± 13.89		│c,d│>│a,b│
The first peak (Nm/kg) of knee adduction moment	0.68 ± 0.1	0.4 ± 0.06	0.64 ± 0.09	0.53 ± 0.05		a,c > d > b
The second peak of knee adduction moment (Nm/kg)	0.68 ± 0.1	0.46 ± 0.02	0.62 ± 0.04	0.5 ± 0.04		a,c > d > b
Knee adduction impulse (Nm.ms/kg)	249.57 ± 72.99	148.8 ± 34.41	250.88 ± 52.33	208.76 ± 58.15		a,c > d > b

The hip moment parameters of the healthy side.

The hip moment parameters are tested at the healthy side in single crutch, double crutches, and prosthetic groups (n = 13), Gait parameters are tested at the right side in healthy group (n = 13); all results in comparison have significance (*P* < .01).

The double-crutches group, all comparisons were significantly lower than those of the other 3 groups, except for hip peak extension moment and hip extension impulse.

the joint moment of prosthetic group was most similar to the healthy group compared to the 2 crutch groups, The hip joint moment parameters were also very similar to those of the healthy group, with the exception of hip peak flexion moment (0.58 ± 0.09).

The knee joint moment curves of the single-crutch group were significantly higher than those of the healthy group at both the sagittal and frontal planes. Most of the knee moment parameters (Table 2) in the single-crutch group were also higher than those in the healthy group. Specifically, the knee moment parameters in the single-crutch group were the highest among the 4 groups, except for the knee peak extension moment and knee extension impulse.

In contrast, the double-crutches group showed significantly lower knee moment values compared to the other 3 groups.

For the prosthetic group, the knee joint moment curve was significantly higher than that of the healthy group at the frontal plane. Additionally, most knee moment parameters in the prosthetic group were significantly higher than those in the healthy group, except for the knee peak extension moment and knee extension impulse.

### 3.3. Joint contact force

**Figure 4.**
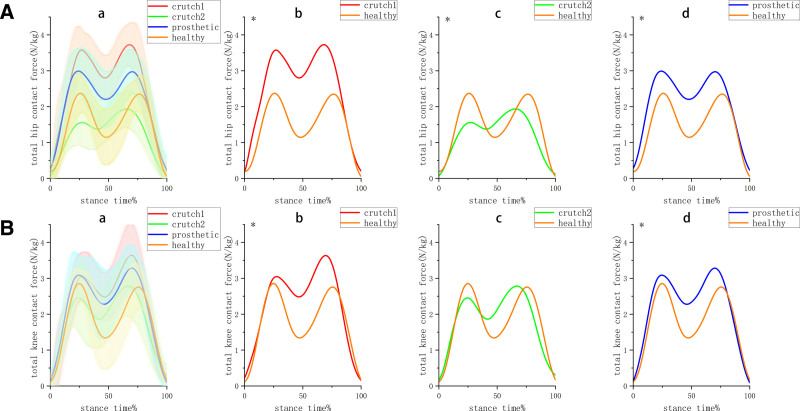
Curves of total joint contact force (n = 13); crutch1 is the single-crutch group, crutch2 is the double-crutches group, and prosthetic is the prosthetic group, healthy is the healthy group. (A) total hip contact force (B) total knee contact force. (a) comparison among 4 groups (b) comparison between single-crutch group and healthy group (c) comparison between double-crutches group and healthy group (d) comparison between prosthetic group and healthy group, *means *P* < .01.

The single-crutch group and prosthetic group (Fig. 4) exhibited significantly higher hip and knee contact forces compared to the healthy group. Moreover, the joint contact force parameters of the single-crutch group and prosthetic group were significantly higher than those of the healthy group, especially the single-crutch group.

The double-crutches group had the most similar total knee contact forces (Table 3). Its first peak (1.7 ± 0.58 N/kg) and second peak (2.07 ± 0.52 N/kg) of total hip contact force were lower than those of the healthy group (first peak: 2.49 ± 0.44 N/kg; second peak: 2.54 ± 0.54 N/kg).

**Table 3:** The joint contact force parameters of the healthy side.

		Single crutch (a)	Double crutches (b)	Prosthetic (c)	Healthy (d)	Comparison
hip contact force	The first peak (N/kg)	3.96 ± 0.41	1.7 ± 0.58	3.27 ± 0.51	2.49 ± 0.44	a > c > d > b
	The second peak (N/kg)	3.92 ± 0.55	2.07 ± 0.52	3.2 ± 0.54	2.54 ± 0.54	a > c > d > b
	Impulse of hip contact force (N.ms/kg)	189.07 ± 21.04	86.39 ± 20.76	169.05 ± 51.54	100.44 ± 26.6	a,c > d,b
knee contact force	The first peak (N/kg)	3.17 ± 0.78	2.71 ± 0.89	3.43 ± 0.45	3.03 ± 0.28	c > d,b
	The second peak (N/kg)	3.65 ± 0.9	2.88 ± 0.84	3.41 ± 0.61	2.93 ± 0.51	a > d,b
	impulse of knee contact force (N.ms/kg)	167.96 ± 35.54	128.79 ± 43.87	183.38 ± 65.09	115.57 ± 21.69	a,c > d,b

The joint contact force is tested at the healthy side in single crutch, double crutches and prosthetic groups (n = 13), Gait parameters are tested at the right side in healthy group (n = 13); all results in comparison have significance (*P* < .01).

## 4. Discussion

The main findings of the present trial is that the load on the healthy limb with a prosthetic device was closest to that of the healthy control group among all 3 groups.

### 4.1. Prosthetic

In the experiment, patients were allowed to choose their preferred walking speed, which enabled observation of their movement abilities when using different walking assistive devices. Among these 4 groups, the healthy control group exhibited the fastest walking speed. Although the prosthetic group’s speed was not as fast as the healthy group, it was still better than the single-crutch and double-crutches groups. This result aligns with previous research on the energy cost of gait patterns when comparing prosthetics and crutches in transtibial amputees,^[16]^ the experiment showed that compared to using crutches, the prosthetic group had a 21% increase in VO^2^ uptake rate and a 92% increase in EE per minute. These findings indicate that while assistive devices can enhance support points and contact areas, upper limb support cannot fully replace lower limb support in human gait. The circumference and positioning of the lower limbs play a crucial role in providing favorable support and stability. Therefore, when selecting and designing assistive devices, prioritizing lower limb support is essential.

Although prosthetics outperform single crutches and double crutches in terms of mobility, there is still a gap between the prosthetic group and healthy control groups. In this study, the prosthetic group exhibited hip joint moment and knee flexion-extension moment similar to the healthy group, but the knee adduction moment was higher in the prosthetic group. The 2 peak points of adduction torque are sensitive indicators in osteoarthritis, the prolonged high adduction torque can lead to cartilage wear and narrowing of joint spaces,^[17]^ contributing to the development of osteoarthritis.^[18]^ Multiple research reports have indicated that prosthetic users are at a higher risk of developing knee osteoarthritis,^[12,13]^ which emphasizes the importance of optimizing prosthetic design and rehabilitation training by reducing knee adduction moment on the intact limb. Both the peak points and the overall integral of the adduction moment should be minimized.

Besides knee joint adduction torque, the prosthetic group also exhibits higher joint contact forces at both the hip and knee joints compared to the healthy control group. For individuals without amputations, higher joint torques are typically associated with increased joint contact forces, as greater torque requires more force to maintain stability and resist joint movement.^[19]^ Notably, in this experimental results, while hip joint torque as an output force did not significantly differ between the prosthetic and healthy groups, the internal hip joint contact force was notably higher in the prosthetic group. This suggests that increasing internal hip joint contact force on the intact side contributes to enhancing joint stability and maintaining balance.

Based on the comparison of 3 assistive walking devices with the healthy control group, bionic prosthetics emerge as the most recommended long-term option for above-knee amputees. This experiment found that, apart from the knee adduction moment, other joint moments did not significantly differ from those of the healthy control group. This suggests that the elastic storage-release design of mechanical prosthetics can meet the dynamic requirements for above-knee amputees’ walking.^[20,21]^

### 4.2. Crutch

In this experiment, the walking speed of the single-crutch group was significantly slower than that of the healthy control group, and it is the slowest among all 3 walking assistive devices. The single-crutch gait exhibited short strides and a high-frequency hopping pattern. This may be due to the crutch’s support point being higher than the intact limb, making it challenging for both limbs to coordinate effectively. Additionally, the single-crutch group had the highest joint moments and joint contact forces among all 3 assistive walking devices. Therefore, using a single crutch has a high proportion with significant stress concentration^[22]^ and an increased risk of falling.^[23]^

In contrast, the double-crutches group had significantly lower joint moments compared to the healthy group, making it the lowest among all 3 assistive devices. Insufficient joint torque implies reduced muscle contraction. This phenomenon may be attributed to the double crutches relying heavily on upper limb support. Since the upper limbs exert pressure on the crutches to generate forward propulsion, the involvement of the lower limbs is minimal.^[24]^ Unfortunately, this is not conducive to postoperative functional recovery^[25]^ and may lead to muscle atrophy.^[26]^ meanwhile, the double crutch group exhibited knee joint contact forces closest to those of the healthy group, with hip joint contact forces even lower than the healthy group. This symmetry and stability result from the dual upper limb support. Other studies have also reported increased gait symmetry after using crutches.^[27]^

Crutches are assistive devices with specific clinical applications^[28]^ that are capable of unloading foot load. When selecting such devices, it is advisable to prioritize those with more support points and paired crutch supports. Additionally, since these devices unload foot load, they should not be used long-term to avoid adverse adaptations such as muscle atrophy^[5]^ and decreased bone density.^[4]^ To maintain normal musculoskeletal characteristics, incorporating resistance training is recommended when using such devices.

### 4.3. Limitation

This study has some limitations. The walking speed of the patients was not controlled as a variable to pursue natural walking. Speed should be used as a control variable in future experiments to compare the patient’s gait with normal gait and quantify the optimization goal. Additionally, due to the limited number of experimental subjects, postoperative time was not used as a control variable, which could influence the accuracy of the results to some extent.

## 5. Conclusion

This study aimed to investigate the effect of different assistive walking devices for above-knee amputees on the limb load of the healthy side, particularly in comparison to the healthy control group. this findings suggest that bio-inspired prosthetics are the optimal choice for long-term use. However, further optimization of joint contact force and knee adduction moment should be achieved through rehabilitation methods and prosthetic modifications. When using crutches as assistive walking devices, prolonged usage should be avoided, and crutch optimization should be made to minimize stress concentration. Resistance training is a preferable rehabilitation method when using crutches. this experimental design and results analysis were committed to improving secondary chronic conditions in postamputee patients. These findings may provide valuable insights for researchers and clinicians, aiding in the selection and optimization of assistive devices and the design of rehabilitation programs.

## Acknowledgments

We would like to acknowledge the individuals who participated in this study, the staff members of the West China Hospital for their important contribution.

## Author contributions

**Conceptualization:** Lingjie Zeng, Yitian Wang, Yong Nie, Chongqi Tu.

**Data curation:** Lingjie Zeng, Yitian Wang, Minxun Lu.

**Formal analysis:** Lingjie Zeng, Minxun Lu, Yong Nie, Chongqi Tu.

**Funding acquisition:** Yitian Wang, Yong Nie, Chongqi Tu.

**Investigation:** Lingjie Zeng, Li Min.

**Methodology:** Lingjie Zeng, Minxun Lu, Xiangdong Zhu.

**Project administration:** Yong Zhou, Xiangdong Zhu.

**Resources:** Yitian Wang, Yong Zhou.

**Supervision:** Xiangdong Zhu.

**Validation:** Lingjie Zeng, Xiangdong Zhu.

**Visualization:** Lingjie Zeng, Xiangdong Zhu.

**Writing – original draft:** Lingjie Zeng, Chongqi Tu.

**Writing – review & editing:** Lingjie Zeng, Yitian Wang, Minxun Lu, Yong Nie, Yong Zhou, Li Min, Chongqi Tu.
